# Fractalkine, sICAM-1 and Kynurenine Pathway in Restrictive Anorexia Nervosa–Exploratory Study

**DOI:** 10.3390/nu13020339

**Published:** 2021-01-24

**Authors:** Ewa Dudzińska, Kinga Szymona, Renata Kloc, Tomasz Kocki, Paulina Gil-Kulik, Jacek Bogucki, Janusz Kocki, Roman Paduch, Ewa M. Urbańska

**Affiliations:** 1 Chair of Public Health, Medical University of Lublin, 20-090 Lublin, Poland; ewa.dudzinska@umlub.pl; 2 Department of Psychiatry, Psychotherapy and Early Intervention, Medical University of Lublin, 20-439 Lublin, Poland; kinga.szymona@umlub.pl; 3 Laboratory of Cellular and Molecular Pharmacology, Chair and Department of Experimental and Clinical Pharmacology, Medical University of Lublin, 20-090 Lublin, Poland; renata.kloc@umlub.pl; 4 Chair and Department of Experimental and Clinical Pharmacology, Medical University of Lublin, 20-090 Lublin, Poland; tomasz.kocki@umlub.pl; 5 Department of Clinical Genetics, Medical University of Lublin, 20-090 Lublin, Poland; paulina.gil-kulik@umlub.pl (P.G.-K.); janusz.kocki@umlub.pl (J.K.); 6 Department of Organic Chemistry, Faculty of Pharmacy, Medical University of Lublin, 20-090 Lublin, Poland; jacek.bogucki@umlub.pl; 7 Department of Virology and Immunology, Maria Curie-Skłodowska University of Lublin, 20-033 Lublin, Poland; rpaduch@poczta.umcs.lublin.pl; 8 Department of General Ophthalmology, Medical University of Lublin, 20-400 Lublin, Poland

**Keywords:** eating disorders, neuroprotection, neurotoxicity, kynurenine

## Abstract

The link between the kynurenine pathway and immunomodulatory molecules—fractalkine and soluble intercellular adhesion molecule-1 (sICAM-1)—in anorexia nervosa (AN) remains unknown. Fractalkine, sICAM-1, tryptophan (TRP), kynurenine (KYN), neuroprotective kynurenic acid (KYNA), neurotoxic 3-OH-kynurenine (3-OH-KYN), and the expression of mRNA for kynurenine aminotransferases (*KAT1-3*) were studied in 20 female patients with restrictive AN (mostly drug-free, all during first episode of the disease) and in 24 controls. In AN, serum fractalkine, but not sICAM-1, KYNA, KYN, TRP or 3-OH-KYN, was higher; ratios TRP/KYN, KYN/KYNA, KYN/3-OH-KYN and KYNA/3-OH-KYN were unaltered. The expression of the gene encoding *KAT3*, but not of genes encoding *KAT1* and *KAT2* (measured in blood mononuclear cells), was higher in patients with AN. In AN, fractalkine positively correlated with TRP, while sICAM-1 was negatively associated with 3-OH-KYN and positively linked with the ratio KYN/3-OH-KYN. Furthermore, TRP and fractalkine were negatively associated with the body mass index (BMI) in AN. Expression of *KAT1*, *KAT2* and *KAT3* did not correlate with fractalkine, sICAM-1 or BMI, either in AN or control. Increased fractalkine may be an independent factor associated with the restrictive type of AN. Excessive physical activity probably underlies increased expression of *KAT3* observed among enrolled patients. Further, longitudinal studies on a larger cohort of patients should be aimed to clarify the contribution of fractalkine and *KAT3* to the pathogenesis of AN.

## 1. Introduction

Anorexia nervosa (AN) is an eating disorder characterized by a prominent fear of gaining weight resulting in an extreme self-starvation and widespread organ dysfunction. In consequence, the mortality rate in AN is the highest among psychiatric conditions [1]. The overall occurrence of AN in population is stable, between 0.3% for men and 1.0% for women. However, within the last decades, an increased occurrence of AN in the high risk-group of 15–19-year-old girls has been observed [2,3]. The disease still presents a great therapeutic challenge, due to the lack of efficacious treatment [4]. The complex etiology of AN involves genetic, developmental, psychological and socio-cultural factors. Alterations of the hypothalamic–pituitary–adrenal (HPA) axis, changes in the neurotransmitter systems and immune dysfunction have all been linked to the disturbed regulation of food intake in AN [4,5,6]. The contribution of altered immune response to the pathogenesis of AN is an attractive hypothesis; however, the available data are rare and the results are often contradictory [7,8]. The dysregulation of the cytokine profile in AN is supported by the observations of an altered proportion between pro- and anti-inflammatory cytokines in the blood of patients. Interestingly, some, but not all, of the pro-inflammatory molecules seem to be down-regulated during an acute phase of the disease [8,9,10]. Recently, lower levels of nineteen and higher levels of six inflammation-related molecules, including various cytokines, were detected in acute AN, but not during recovery period [11].

The impact of chemokines, a family of chemoattractant cytokines, on brain function is well substantiated [12,13]. Chemokines have been shown to affect the synaptic transmission, plasticity, neurogenesis and neuron-glia communication [14]. The modulation of intercellular adhesion represents an important aspect of their action. The transmembrane chemokine (C-X3-C motif) ligand 1 (CX3CL1, fractalkine) affects the immune response through the G protein-coupled CX3CR1 receptor and acts as an adhesion molecule [14]. Under physiological conditions, fractalkine is constitutively expressed in neurons, whereas glial cells may generate ample quantities of the compound during inflammation. Fractalkine is either membrane-bound, linked with its CX3CR1 receptor, or cleaved to generate a soluble form, able to act distantly [15,16,17]. The compound has been shown to suppress the immune response and microglia-related neurotoxicity, to control axonal and synaptic development and to influence the serotonergic, glutamatergic and GABAergic neurotransmission [15,16]. Pro-inflammatory cytokines and chemokines may also alter the expression of intercellular adhesion molecule-1 (ICAM-1), a transmembrane immunoglobulin-like glycoprotein [18,19]. A soluble ICAM (sICAM-1) is cleaved from its membrane-bound form and circulates in the serum and other body fluids, yet at a relatively low levels [18]. Under pathological conditions, levels of sICAM-1 increase, and the compound appears to have a prognostic value [18,20]. A higher peripheral sICAM-1 level may reflect the progressing severity of cardiovascular disease, major depression, bipolar disorder or dementia [18,20].

Approximately 90% of body tryptophan is catabolized along the so-called kynurenine (KYN) pathway, in the periphery and in the brain [21,22,23]. The KYN pathway is widely accepted as an important link between a disturbed immune response, aberrant brain function and psychiatric disorders [21,23]. KYN, transported into the brain or synthesized in situ, is subsequently converted to several neuroactive metabolites along two major metabolic branches. One arm of the path leads to the synthesis of neuroprotective kynurenic acid (KYNA) [22,23]. KYNA is a unique molecule, able to block the glycine site of the *n*-methyl-D-aspartate (NMDA) receptor complex and the α7- nicotinic cholinergic receptors to stimulate the G protein-coupled receptor 35 (GPR35) and to bind aryl hydrocarbon receptors (AHR) [24]. At nanomolar levels, KYNA affects the release of glutamate, dopamine and GABA, and modulates neurotransmission [25,26,27]. The other arm of the KYN pathway yields cytotoxic compounds, such as highly reactive 3-hydroxy-kynurenine (3-OH-KYN), yielding free radicals, and quinolinic acid, an agonist of NMDA receptors, and a potent excitotoxin, as shown under in vitro and in vivo conditions [21,23]. The balance between protective and neurotoxic kynurenines can be shifted during chronic stress and inflammation. Upon stimulation of the HPA axis, the excessively released cortisol and pro-inflammatory mediators accelerate the metabolic conversion of TRP [28,29,30]. Clinical data consistently reveal an altered function of the central and peripheral KYN pathway in psychiatric disorders [31,32,33,34]. Hence, the role of the aberrant TRP/KYN pathway in the pathogenesis of affective and psychotic diseases is broadly accepted [23].

However, knowledge of the TRP/KYN pathway in AN is very limited, and the available results are unclear. In animals, experimental supplementation with TRP either inhibited stress-induced anorexia or did not alter anxiety-like behavior but elicited depression like-behavior in the forced swim test [35]. Scarce clinical data show that administration of TRP exacerbates the anxiety among AN patients, whereas during re-feeding, an increase in the ratio between TRP and large neutral amino acids is linked with a decrease in depressive symptoms [36,37]. To our knowledge, just a single study has been undertaken to analyze the cerebrospinal fluid (CSF) levels of TRP and its metabolites in AN [38], and the potential associations of peripheral TRP metabolism with immunomodulatory molecules—fractalkine and sICAM-1—remain unknown.

We hypothesized that the balance between neuroprotective KYNA and neurotoxic kynurenines would be changed in AN, and that fractalkine and sICAM-1 may be involved in this process. The correlations of serum fractalkine and sICAM-1 with the expression of mRNA for KYNA biosynthetic enzymes; kynurenine aminotransferases (KAT1-3); and with serum levels of four major metabolites of TRP/KYN pathway, i.e., TRP, KYN, neuroprotective KYNA, and neurotoxic 3-OH-KYN, were studied in a cohort of female patients with restrictive type of AN.

## 2. Materials and Methods

### 2.1. Study Population

The studied cohort included 20 female patients with AN (age 18.97 ± 2.54 years), diagnosed according to the ICD-10 and DSM-5 diagnostic criteria, and 24 healthy female individuals (age 19.15 ± 2.57 years). Patients were recruited at the Department of Psychiatry, Psychotherapy and Early Intervention Medical University in Lublin (*n* = 19) and from the Mental Health Outpatient Clinic of Children’s University Hospital in Lublin (*n* = 4), during the years 2016–2018. The control individuals were recruited among high school students in Lublin area and from students of Medical University in Lublin during the same period of time. Exclusion criteria included any current physical disorders, history of neurological, immune-mediated or any other chronic disorders, and CRP > 3.0 mg/L (measured on the day of blood sampling). Control subjects were screened for the psychiatric disorders, any medical problems and history of any chronic disease.

Based on the oral interview with patients and their family members, enrolled patients manifested morbid fear of gaining weight; excessive, daily physical exercise lasting minimum 8 h/day; had not regularly engaged in binge-eating or purging within last 3 months; and were further classified to a restrictive type of AN. Height and body weight were measured on the day of blood sampling, and, from these measurements, the body mass index (BMI; kg/m^2^) was calculated. All of the control patients were menstruating regularly, while all of the enrolled AN patients had amenorrhea. The studied cohort of patients was during first episode of AN (an average duration of disease 9.5 ± 4.6 months) and basically drug-free. Only three patients used drugs: sertraline (*n* = 1) and olanzapine (*n* = 2). Other treatment was based on the cognitive-behavioral therapy and supervised weight gain.

The study was approved by the Local Ethical Committee of the Faculty of Medicine at the Medical University, Lublin, number KE-0254/77/2012, and was in accordance with the Helsinki Declaration of 1975. All participants were informed about the aim and protocol of the study and gave the written informed consent. Clinical and biological characteristics of patients with AN and control subjects are shown in Table 1.

### 2.2. Genetic Analyses

Blood samples (2 × 5 mL) were taken from all the subjects by venipuncture and collected into an EDTA vacuum tube between the hours of 6:00 a.m. and 9:00 a.m. Samples for measurements of metabolites levels were centrifuged at 4000 g for 10 min within 1 h. Resulting plasma was withdrawn and stored at −72°C until further analyses. Peripheral blood mononuclear cells for genetic analyses were isolated by the Ficoll-paque density gradient method. Measurement of mRNA transcript levels for *KAT1* (glutamine transaminase K/cysteine conjugate beta-lyase; *CCBL1*), *KAT2* (aminoadipate aminotransferase; *AADAT*) and *KAT3* were assessed in isolated lymphocytes, by TaqMan real-time polymerase chain reaction (PCR). Gene-specific probes (Hs00187858_m1 for *CCBL1*, Hs00212039_m1 for *AADAT*, Hs00219725_m1 for *KAT3*, and Hs99999905_m1 for *GAPDH*) were obtained from Applied Biosystems. As a reference gene, *GAPDH* was used. Only the samples that showed the presence of PCR product for the *GAPDH* gene were included in the analysis. The results were analyzed using Expression Suite v. 1.0.3 software (Life Technologies). The gene expression value (RQ) *of AADAT, CCBL1* and *KAT3*, relative to *GAPDH* value, was calculated by the formula RQ = 2^−ΔΔCt^ [39].

### 2.3. Chromatographic Analyses of TRP Metabolites

Serum KYNA, KYN and TRP levels were determined by the ultra-high pressure liquid chromatography (UHPLC) method (Waters Acquity UHPLC system; Waters C18 analytical column), as described before [40]. Briefly, the mobile phase containing 20 mM sodium acetate, 3 mM zinc acetate and 7% acetonitrile was run with the flow rate of 0.1 mL/min. Quantification of TRP and its metabolites was performed by a UV variable wavelength detector (KYN at 365 nm; TRP at 250 nm) and by a fluorescence detector (KYNA-344 nm excitation and 398 nm emission). 3-OH-KYN levels were determined fluorometrically with an electrochemical detector (potential of working electrode: + 0.20 V; Coulochem III, ESA), as described before [28]. The HPLC column (HR-80; 3µm; C18 reverse-phase column; ESA) was perfused at 0.6 mL/min using a mobile phase consisting of 2% acetonitrile, 0.9% triethylamine, 0.59% phosphoric acid, 0.27 mM sodium EDTA and 8.9 mM heptane sulfonic acid.

### 2.4. Fractalkine and sICAM-1 Assay

The levels of fractalkine and of human sICAM-1/CD54 were measured immunoenzymatically (ELISA) using a commercially available kit (R&D Systems, Inc., Minneapolis, MN, USA), according to the manufacturer’s instruction. The optical density of the end product was determined using a microplate reader (model EL800, Biotek, Winooski, VT, USA) at 450 nm and analyzed with KC Junior program. Sensitivity of fractalkine test was 0.072 ng/mL, while sICAM-1/CD54 was 0.254 ng/mL.

### 2.5. Statistical Analyses

Differences in the expression of genes, levels of serum fractalkine, sICAM-1, TRP, KYN, KYNA, 3-OH-KYN and metabolites ratios between any two groups of the subjects were evaluated using Mann–Whitney U-test. Correlations between variables were evaluated using Spearman’s correlation analyses and are presented by correlation coefficients (R). *p*-Value < 0.05 was considered statistically significant. All the analyses were performed with the use of Statistica v.13 program.

## 3. Results

Serum fractalkine level was significantly higher among AN patients than among healthy control (*p* < 0.001) (Table 2). There were no statistically significant differences in the levels of sICAM-1, KYNA, KYN, TRP and 3-OH-KYN in AN patients and controls (Table 2). The ratios TRP/KYN, KYN/KYNA, KYN/3-OH-KYN and KYNA/3-OH-KYN in AN group were not changed vs. control (Table 2). The expression of gene encoding *KAT3*, but not of genes encoding *KAT1* and *KAT2*, was significantly higher in AN group than in control (*p* < 0.001) (Table 2).

In AN group, but not in control, fractalkine level significantly, positively correlated with TRP level (*p* < 0.01) (Table 3). Also in AN, but not in control, sICAM-1 level was significantly, negatively associated with serum 3-OH-KYN (*p <* 0.05), and was positively linked with the ratio L-KYN/3-OH-KYN (*p <* 0.05) (Table 3).

In AN, but not in control, TRP and fractalkine were negatively associated with BMI (both *p* < 0.05) (Table 4).

There were no significant correlations of genes encoding *KAT1*, *KAT2* and *KAT3* with fractalkine, sICAM-1 or BMI, either in AN or in control (Table 5).

## 4. Discussion

In the studied cohort of patients during first episode of restrictive AN, serum levels of soluble fractalkine, but not of sICAM-1, TRP, KYN, KYNA and 3-OH-KYN, were higher than in control individuals. A significant, positive correlation of fractalkine with TRP and a negative correlation of TRP and fractalkine with BMI were found in AN patients. Furthermore, a negative correlation of sICAM-1 with serum 3-OH-KYN and a positive correlation of sICAM-1 with the ratio KYN/3-OH-KYN were also detected among AN patients.

The research on TRP and other kynurenines in AN is scarce, and the available results are contradictory. Among patients studied here with a restricting type of AN, serum TRP level did not differ from control. Supplementation with TRP was proposed as a therapeutic approach, aimed to increase brain serotonin levels and thus to reduce the depressive symptoms in AN [35,41]. Indeed, it was shown that the reduction of anxiety and depression correlated with the ratio between serum TRP and large neutral amino acids in AN patients [36]. Contrasting data were obtained in a group of AN patients given an intravenous administration of TRP. This procedure blunted the growth hormone secretion and increased the anxiety level among AN patients [37]. Furthermore, a dietary-induced reduction of serum TRP level was associated with a decreased anxiety among AN patients, as shown by others [42]. The possible outcome of manipulating TRP level in AN is therefore unclear.

To our knowledge, only one study evaluated the fate of TRP metabolism along the KYN pathway in AN. The analyses, in contrast to our paradigm, were carried out in the cerebrospinal fluid (CSF) [38]. A decrease of CSF KYNA, and no change in the levels of TRP and KYN, were found in a small (*n* = 10) group of medication-free female patients with AN [38]. In the brain, KYNA is produced mainly within astrocytes, primarily by KAT2 [43]. Interestingly, in the animal model of AN, the density of astrocytic cells is dramatically reduced [44], which could underlie lower central KYNA formation.

In the studied group, the peripheral levels of L-KYN, KYNA and 3-OH-KYN were unaltered compared to control. There are two possible explanations for this observation. First, revealed by other authors, decrease of CSF KYNA level [38] may be region-specific and restricted to the brain. Furthermore, peripheral formation of KYNA may be affected differently and influenced by various factors able to restore KYNA levels to normal values.

Interestingly, in the experimental model of AN, animals exposed to additional physical exercise during re-feeding period manifested an increased expression of muscle *KAT3* and *KAT4* mRNA levels, in contrast to the re-fed mice without access to running wheel [45]. Moreover, muscle conversion of KYN into KYNA was stimulated during recovery from malnourished state, but only in animals subjected to physical activity [45]. Physical exercise was also shown to stimulate the expression of muscle *KAT1*, *KAT3* and *KAT4* in humans [46].

Similarly, we detected a significantly higher expression of *KAT3* gene in AN patients than in control. Enrolled patients were using excessive daily exercise as a method for losing weight, prior to the initiation of therapeutic regimen. Hence, it is very likely that the observed increase of *KAT3* expression is a consequence of their intense physical activity. This, in turn, may overcome an initial deficit of KYNA synthesis. Further, longitudinal studies on a larger cohort of patients, including restrictive and binge-eating/purging types of AN, should be performed in order to clarify this intriguing possibility.

Patients studied here with AN had significantly higher serum level of fractalkine than control individuals. To our knowledge, there is only a single report about fractalkine in AN. In a cohort of 34 young Chinese patients with AN, the level of fractalkine was lower than in control [47]. The difference between our studies may be attributed to the average age of enrolled patients, 12–18 years [47] vs. 15–24 years in our study. Furthermore, in above-mentioned study, the AN group was not divided into restrictive and binge-eating/purging subtypes. After adjusting for BMI, fractalkine concentrations turned out to be statistically higher in AN than in control, and, similarly to our findings, a correlation of fractalkine with BMI was detected [47]. The authors suggested that higher fractalkine may reflect the presence of chronic inflammation among patients with AN [47]. Bearing in mind that the exclusion criteria used here included active inflammatory disease or CRP level higher than 3.0 mg/L, the observed change in fractalkine does not seem to be associated with ongoing inflammation.

In the absence of brain injury, the fractalkine/CX3CR1 axis may reduce the microglial activation and regulate proper neurogenesis and memory processes. Conversely, during inflammation, fractalkine was shown to shift the microglial cells and astrocytes into activated state and to act proinflammatory [48,49,50]. The compound plays an important role in the crosstalk between neurons, microglia and astrocytes, yet its contribution to mood disorders is not fully clarified. As shown experimentally, hippocampal and cortical expression of fractalkine and CX3CR1 increases among adult rats susceptible to chronic mild stress, in contrast to stress-resilient animals [51]. Correspondingly, CX3CR1^−/−^ knockout mice are resistant to chronic stress-induced mood alterations [52]. Prenatal stress seems to affect the fractalkine axis diversely since in an animal model of depression based on the prenatal stress procedure, the hippocampal and cortical levels of CX3CL1 and CX3CR1 are diminished and return to normal values during therapy with antidepressants [13].

Among enrolled patients with a restrictive type of AN, a positive correlation of TRP with fractalkine was shown. Moreover, the severity of disease reflected by the BMI was inversely correlated with TRP and fractalkine serum levels. Increased level of fractalkine may, therefore, constitute an independent factor associated with restrictive type of AN. Such conclusion corresponds with the data indicating that higher serum fractalkine is linked with an augmented sensitivity towards stressful stimuli and may negatively affect the mood [16,52,53]. A relatively low sensitivity of fractalkine assay used here is a limitation of the study; however, we were able to detect the compound in all but three patients’ samples. Whether high serum fractalkine precedes the development of AN or is a marker of the disease remains to be established. Further, longitudinal studies are needed to assess the potential link of fractalkine levels with the neuropsychological deficits in the course of disease.

Analyses of the KYN pathway in fractalkine receptor deficient mice (CX_3_CR1^−/−^) revealed that the stimulation with lipopolysaccharide evokes 2–3 fold stronger induction of indoleamine 2,3-dioxygenase (IDO), an enzyme degrading TRP to KYN, and kynurenine monooxygenase (KMO), metabolizing KYN to 3-OH-KYN, in CX3CR1^−/−^ mice than in the CX3CR1^+/−^ control [54]. We have initially hypothesized that fractalkine may influence the conversion of TRP along the KYN pathway and correlate with the serum levels of KYNA, KYN, 3-OH-KYN or the ratios between these metabolites in AN. Nonetheless, data presented here do not support this assumption.

An increase of blood sICAM-1 was reported in a number of psychiatric conditions, such as major depression, bipolar disorder or dementia [18]. However, unchanged levels of serum sICAM-1 were detected in patients with AN, despite the increase of vascular cell adhesion molecule (VCAM)-1 concentration [55,56]. In contrast, others reported an increase of serum sICAM-1 in the group of 15 young female patients with AN [57]. Among patients enrolled here with the first episode of restrictive AN, serum level of sICAM-1 remained within control values; however, sICAM-1 was significantly, negatively associated with serum 3-OH-KYN and positively correlated with the ratio KYN/3-OH-KYN. These findings suggest the potential ability of sICAM-1 to shift the KYN pathway away from the neurotoxic branch, however, we did not observe any changes in the level of 3-OH-KYN.

## 5. Conclusions

Despite no changes in the serum levels of neuroprotective KYNA and neurotoxic kynurenines among patients with the first episode of restrictive type of AN, an increase of *KAT3* expression, most probably linked to the excessive physical activity of AN patients, occurred. Increased level of fractalkine may constitute an independent factor associated with restrictive type of AN; however, the temporal course of fractalkine changes requires detailed studies. Further research should aim to clarify the possibility of modulation of the TRP/KYN pathway by fractalkine and sICAM among AN patients.

## Figures and Tables

**Table 1:** Demographic characteristics of control subjects and patients with anorexia nervosa (AN).

Variable	Control	Anorexia Nervosa	*p*-Value
	Mean ± SD	Mean ± SD	
Age (years)	19.15 ± 2.57	18.97 ± 2.54	0.781
Body mass (kg)	52.10 ± 1.89	39.18 ± 1.3	<0.001
BMI (kg/m^2^)	20.07 ± 0.93	15.17 ± 0.92	<0.001
Disease duration (months)	-	9.5 ± 4.6	

BMI, body mass index.

**Table 2:** Fractalkine, soluble intercellular adhesion molecule-1 (sICAM-1), tryptophan (TRP) metabolites and expression of kynurenine aminotransferases (KAT1-3) in anorexia nervosa.

Variable	Control	Anorexia Nervosa	*p*-Value
Fractalkine (ng/mL)	0.059 ± 0.003	**0.066 ± 0.006**	<0.001
sICAM-1 (ng/mL)	182.710 ± 23.76	178.44 ± 40.94	0.776
TRP (µmol/L)	42.85 ± 10.35	41.11 ± 13.58	0.456
KYN (µmol/L)	2.21 ± 0.91	2.26 ± 0.60	0.591
KYNA (nmol/L)	38.09 ± 8.81	42.22 ± 15.17	0.704
3-OH-KYN (nmol/L)	12.63 ± 13.59	20.07 ± 23.49	0.225
Ratio			
TRP/KYN	26.52 ± 30.79	18.79 ± 5.44	0.454
KYN/KYNA	0.058 ± 0.02	0.056 ± 0.02	0.391
KYN/3-OH-KYN	2.82 ± 1.56	3.35 ± 3.61	0.583
KYNA/3-OH-KYN	46.61 ± 23.57	61.05 ± 72.35	0.675
logRQ *CCBL1* (*KAT1*)	−0.176 ± 0.362	0.006 ± 0.288	0.127
logRQ *AADAT (KAT2*)	−0.037 ± 0.39	−0.002 ± 0.42	0.551
logRQ *KAT3*	−0.295 ± 0.303	**0.057 ± 0.168**	<0.001

Values statistically different from controls (*p*-values < 0.001) are marked in bold.

**Table 3:** Correlations of fractalkine and sICAM-1 with TRP metabolites in anorexia nervosa.

	Control				Anorexia Nervosa			
	Fractalkine		sICAM-1		Fractalkine		sICAM-1	
	R	*p*-Value	R	*p*-Value	R	*p*-Value	R	*p*-Value
TRP	−0.219	0.357	0.412	0.071	**0.679**	<0.01	0.007	0.977
KYN	0.001	0.993	0.063	0.791	0.245	0.359	0.327	0.185
KYNA	−0.063	0.785	0.321	0.166	0.381	0.144	−0.089	0.723
3-OH-KYN	−0.036	0.904	0.350	0.167	0.167	0.463	**−0.464**	<0.05
Ratio								
TRP/KYN	−0.162	0.482	0.204	0.387	0.324	0.238	−0.144	0.579
KYN/KYNA	0.149	0.518	−0.174	0.462	−0.194	0.488	0.414	0.098
KYN/3-OH-KYN	−0.157	0.532	−0.203	0.433	0.195	0.468	0.498	0.05
KYNA/3-OH-KYN	0.054	0.829	−0.04	0.852	0.217	0.418	**0.457**	<0.05

Values statistically different from controls (*p*-values < 0.05) are marked in bold. Correlation coefficient – R.

**Table 4:** Correlations of fractalkine, sICAM-1 and TRP metabolites with BMI in anorexia nervosa.

	Control		Anorexia Nervosa	
	R	*p*-Value	R	*p*-Value
Fractalkine	0.214	0.338	−**0.586**	<0.05
sICAM-1	−0.084	0.716	0.034	0.889
TRP	−0.117	0.602	−**0.518**	<0.05
KYN	−0.020	0.926	−0.401	0.088
KYNA	−0.128	0.567	−0.379	0.108
3-OH-KYN	0.001	0.993	−0.202	0.391

Values statistically different from controls (*p*-values < 0.05) are marked in bold.

**Table 5:** Correlations of fractalkine, sICAM-1 and BMI with *CCBL1, AADAT* and *KAT3* in anorexia nervosa.

	*AADAT*		*CCBL1*		*KAT3*	
	R	*p*-Value	R	*p*-Value	R	*p*-Value
**Control**						
BMI	0.289	0.243	0.280	0.195	0.459	0.027
Fractalkine	0.350	0.168	0.124	0.579	−0.05	0.809
sICAM-1	−0.144	0.579	−0.168	0.482	−0.163	0.478
**Anorexia Nervosa**						
BMI	−0.177	0.527	−0.245	0.325	−0.120	0.632
Fractalkine	0.451	0.140	0.182	0.514	−0.026	0.924
sICAM-1	−0.345	0.226	−0.431	0.083	−0.451	0.069

## Data Availability

Supporting data are enclosed as Supplementary Table S1.

## Supplementary Materials

The following are available online at https://www.mdpi.com/2072-6643/13/2/339/s1, Table S1: The expression of genes, levels of metabolites and demographic characteristics of AN patients and controls.
