# Supplementary material for: Fractalkine, sICAM-1 and Kynurenine Pathway in Restrictive Anorexia Nervosa–Exploratory Study
Source: Nutrients. 2021 Jan 24;13(2):339. doi: 10.3390/nu13020339 (PMC7910978; doi:10.3390/nu13020339)
Supplement: Supplementary file 1 [file nutrients-13-00339-s001.pdf]

Sample	RQ AADAT	logRQ AADAT	RQ CCBL1	logRQ CCBL1	RQ KAT3	logRQ KAT3
W07	3,947999954	0,596377139	6,638999939	0,822102665	1,521000028	0,182129222
W15			2,517999887	0,401055706	2,628999949	0,419790578
W21	1,960000038	0,29225608	0,799000025	-0,097453207	0,913999975	-0,039053816
W22	0,182999998	-0,737548914	0,851000011	-0,070070434	1,003000021	0,001300942
W26	1,388000011	0,14238947	0,591000021	-0,228412504	0,741999984	-0,129596104
W27	1,161999941	0,065206106	0,625	-0,204119983	0,779999971	-0,107905413
W28			0,463999987	-0,333482032	0,718999982	-0,143271121
W30	0,526000023	-0,279014237	0,69599998	-0,157390773	1,338000059	0,126456133
W33	2,773999929	0,443106446	2,305000067	0,362670942	1,817000031	0,259354935
W34	1,422000051	0,152899612	1,508999944	0,178689224	1,70599997	0,231979019
W35	2,305999994	0,362859302	1,090000033	0,037426511	1,506999969	0,178113244
W36	0,957000017	-0,019088054	0,93599999	-0,028724156	0,98299998	-0,007446491
W37	1,292999983	0,111598519	0,805000007	-0,094204116	0,930999994	-0,031050322
W38			1,133999944	0,054613033	1,478999972	0,169968166
W39	1,245000005	0,095169353	0,573000014	-0,241845368	0,769999981	-0,113509286
W40	0,843999982	-0,073657563	0,915000021	-0,038578896	1,271999955	0,104487096
W41	0,112999998	-0,946921564	0,495999992	-0,304518331	0,628000021	-0,202040342
W42	0,586000025	-0,232102365	1,128000021	0,052309108	1,353999972	0,131618655
W43						
W44						
KA 17			0,560000002	-0,251811971	0,317999989	-0,497572895
KA 18			2,569000006	0,409764105	0,823000014	-0,084600157
KA 19	1,20599997	0,081347297	1,044999957	0,019116273	0,973999977	-0,011441053
KA 20	3,694999933	0,567614435	2,115999937	0,32551565	2,342999935	0,369772277
KA 21	0,145999998	-0,835647151	0,344999999	-0,462180906	0,524999976	-0,279840716
KA 22	4,252999783	0,628695361	1,70599997	0,231979019	0,846000016	-0,072629629
KA 24	0,589999974	-0,229148008	1,667999983	0,222196042	0,317000002	-0,498940735
KA 23	0,961000025	-0,017276601	0,279000014	-0,554395775	0,090999998	-1,040958616
KA 01	2,829999924	0,451786424	1,442999959	0,159266319	1,462000012	0,164947376
KA 02	2,380000114	0,376576978	1,097000003	0,040206629	0,577000022	-0,23882417
KA 03	0,661000013	-0,179798532	0,5	-0,301029996	0,419	-0,377785977
KA 04	0,95599997	-0,019542121	0,287	-0,542118103	0,671999991	-0,172630733
KA 05	1,057999969	0,024485655	0,219999999	-0,657577322	0,238000005	-0,623423033
KA 06			0,416000009	-0,38090666	0,601000011	-0,22112552
KA 07	0,640999973	-0,193141989	0,456	-0,341035157	0,361999989	-0,441291443
KA 08	1,271000028	0,10414556	1,378999949	0,13956425	0,592999995	-0,22694531

KA 09	0,582000017	-0,235077003	0,088	-1,05551733	0,149000004	-0,82681372
KA 10	1,218000054	0,085647308	0,74000001	-0,130768275	0,425999999	-0,370590402
KA 11	0,308999985	-0,510041541	0,363000005	-0,440093368	0,483999997	-0,315154641
KA 12	0,6600001	−0,176034002	0,783999979	-0,105683949	0,714999974	-0,145693974
KA 13	0,301999986	-0,519993077	0,397000015	-0,401209477	0,462000012	-0,335358013
KA 14			1,09800005	0,04060236	0,412999988	-0,384049961
KA 15	0,556999981	-0,254144819	1,480999947	0,170555043	1,103999972	0,042969063
KA 16			0,416999996	-0,379863949	0,316000015	-0,500312897

Sample	nmol KYNA/L	µmol L-KYN/L	µmol TRP/L	3-OH KYN µmol/100 µL	Ratio TRP/L-KYN	Ratio L-KYN/KYNA	Ratio L-KYN/3OH
W07	40,56256062		42,70643781				
W15	63,03265438	2,408141756	64,54974672	2,48199	26,80479526	0,03820467	0,97024636
W21	66,66666667	2,724856947	47,1814292	2,23766	17,31519493	0,040872854	1,21772608
W22	72,32460394	3,139868216	57,21192151	1,33731	18,2211219	0,043413556	2,34789855
W26		2,539028775	50,73673339		19,98273272		0,30238592
W27	72,35693501	2,862546205	74,85221069	3,80318	26,14882183	0,039561463	0,75267177
W28	46,85418687	2,468988148	37,20356071	3,22202	15,06834317	0,052695145	0,76628579
W30	29,43420627	1,423536491	22,81906261	2,07302	16,0298403	0,048363339	0,68669694
W33	33,21047527	1,825915032	28,64456794	2,2054	15,68778801	0,054980093	0,82792919
W34	38,41577756	1,966818533	34,21433138	2,09402	17,39577434	0,051198197	0,93925489
W35	26,21403168	2,423465479	24,96483549	1,48404	10,3012961	0,09244917	1,63301897
W36	33,57258325	2,457850418	37,4499582	0,81527	15,23687443	0,073210048	3,01476863
W37	32,27287423	1,427348734	39,35228446	0,4644	27,57019607	0,044227506	3,07353302
W38	40,75008083	2,180677907	48,29390646	0,63719	22,14628135	0,053513462	3,42233542
W39	39,76721629	2,820387279	36,00501648	0,80657	12,76598315	0,070922422	3,4967669
W40	31,84610411	1,746381172	33,63645306	0,58232	19,26065947	0,054838142	2,999006
W41	24,69447139	1,3049832	25,22697093	0,13188	19,33126107	0,052845156	9,89523203
W42	35,37019075	1,421443495	39,50277873	0,11691	27,79060784	0,040187612	12,1584423
W43	31,50339476	3,257449759	36,47371268	0,29839	11,19701465	0,10339996	10,9167524
W44	43,3624313	2,565340729		0,60694		0,059160445	4,22667929
KA 17	31,82670546	3,183447389	41,72232332	0,7399	13,10601942	0,100024409	4,30253736
KA 18	41,73294536	2,228368323	49,90606403	0,7399	22,39578777	0,053395903	1,28855832
KA 19	40,48496605	3,048000628	44,1803964	1,72935	14,49487772	0,075287222	0,48682251
KA 20	54,52958293	2,747431408	63,51940196		23,11955879	0,050384237	
KA 21	39,87067572	1,595535938	45,48517189		28,50777022	0,04001778	0,57197919
KA 22	54,08341416	4,517508288	64,28957852	1,77976	14,23120323	0,083528534	1,7320406

KA 24	50,34594245	2,23546956	52,8662765	2,6082	23,64884651	0,044402179	1,12299037
KA 23	51,7038474	3,745865398	46,22485615		12,34023416	0,072448485	2,94833955
KA 01	42,37956676	2,68957501	49,44326956	1,2705	18,38330197	0,063463957	4,85071331
KA 02	26,61493695	2,046277643	26,93896621	0,55447	13,16486367	0,076884557	2,53361932
KA 03	34,46492079	1,534913795	38,34898933	0,80765	24,98445806	0,044535538	2,17440685
KA 04	40,18105399	2,334363636	45,77583239	0,7059	19,60955512	0,058096127	5,3406933
KA 05	35,55124475	1,891096917	30,30541484	0,43709	16,02531027	0,05319355	3,53436422
KA 06	42,65114775	1,297059714	52,8185708	0,53506	40,72177266	0,030410898	2,22457331
KA 07	35,14387326	2,420923983	42,75414351	0,58306	17,66025857	0,068886089	5,62364743
KA 08	28,41254446	1,500454105	32,64348596	0,43049	21,75573771	0,052809565	
KA 09							
KA 10	24,72033624	1,449100946	25,53533664	0,30237	17,62150299	0,058619791	2,5617879
KA 11	38,68089234	2,086493073	44,69581469	0,56566	21,4215016	0,053941182	3,18626393
KA 12	35,84222438	1,653093336	40,84345645	0,65484	24,70728999	0,046121394	2,42144068
KA 13	28,14742968	2,385866295	33,28677519	0,68269	13,95165155	0,084763203	5,17025592
KA 14	32,05302296	1,777850867	35,94944179	0,46146	20,22072969	0,055465934	2,82409236
KA 15	28,56773359	0,217372617	35,0735258	0,62953	161,3520889	0,007609026	
KA 16	0,059068456	2,209094682	41,47899231		18,776466	0,059068456	

Sample	Weight	Age	ICAM 1	Fractalkine	BMI
W07	39,1	17	126,837	0,069	14,22
W15	38	24	140,05	0,071	13,2
W21	39,3	20	164,828	0,071	15,8
W22	38,2	16	188,267	0,065	14,3
W26	38,7	19	164,842	0,07	14,84
W27	41,2	18	213,003	0,063	16,31
W28	39,5	22	140,962	0,066	14,42
W30	40,2	21	170,116	0,06	16
W33	40,7	18	131,481	0,061	15,2
W34	39,9	17	100,204	0,062	17
W35	38,6	16	236,595		15,5
W36	37,5	17	210,809	0,066	14,3
W37	36,8	16	209,655	0,068	15
W38	38,2	15,5	191,417	0,085	14,7
W39	39,7	20	248,192	0,061	15
W40	41,7	18	144,496	0,058	16,44
W41	40,95	22	188,282		16,32

W42	38,7	19			15,1
W43	38,1	23	189,12	0,068	14,8
W44	38,5	21	231,141	0,066	15
KA 17	48,5	26	238,235	0,056	19,42
KA 18	52,2	27		0,062	20,2
KA 19	51,2	25	171,858	0,06	19,88
KA 20	49,9	25	177,368	0,057	19,73
KA 21	52,35	27	224,335	0,058	19,84
KA 22	54,4	27	198,773	0,064	20,85
KA 24	50,7	25	221,867	0,059	19,44
KA 23	51,6	25	137,001	0,061	19,16
KA 01	54,7	20	172,344	0,062	21,28
KA 02	49,7	28	170,652	0,059	21,7
KA 03	50,1	26	179,889	0,064	19,5
KA 04	54,3	27	201,289	0,057	21,54
KA 05	51,2	26,5	183,43	0,063	19,67
KA 06	52,4	25,5	184,497	0,052	18,97
KA 07	53,4	20	179,241	0,053	19,72
KA 08	49,6	22	171,382	0,059	19,33
KA 09	52,9	22	191,172	0,059	20,32
KA 10	50,7	25	176,597	0,064	20,41
KA 11	52,5	23	181,22	0,058	19,37
KA 12	53,6	27	173,427	0,066	20,45
KA 13	52,4	28	152,577	0,061	19,47
KA 14	54,8	27	167,426	0,065	20,2
KA 15	55,2	20	163,554	0,054	21,35
KA 16			180,299	0,059	21,48

Supplementary Table S1. The expression of genes, levels of metabolites and demographic characteristics of AN patients and controls.
